# Risk Factors for Subclavian Vein Thrombosis in Cancer Patients With Total Parenteral Nutrition

**DOI:** 10.14740/jocmr1862w

**Published:** 2014-07-28

**Authors:** Ricardo Berea-Baltierra, Rodolfo Rivas-Ruiz, Elpidia Vela-Martinez, Maria de la Luz Sevilla-Gonzalez, Juan Osvaldo Talavera-Pina, Elena Valencia-Jimenez, Irene Perez-Franco, Laura Escobedo-Hernandez

**Affiliations:** a Department of Internal Medicine and Nutritional Support, Oncology Hospital, Centro Medico Nacional Siglo XXI, Mexico City, Mexico; b Department of Pediatrics, Pediatric Hospital, Centro Medico Nacional Siglo XXI, Instituto Mexicano del Seguro Social, Mexico City, Mexico; c Postgraduate Unit, Escuela Superior de Medicina, Instituto Politecnico Nacional, Mexico City, Mexico; d Centro de Adiestramiento en Investigacion Clinica, Coordinacionde Investigacion en Salud, Centro Medico Nacional Siglo XXI, Instituto Mexicano del Seguro Social, Mexico City, Mexico; e Department of Radiology, Oncology Hospital, Centro Medico Nacional Siglo XXI, Mexico City, Mexico

**Keywords:** Subclavian vein thrombosis, Cancer, Parenteral nutrition, Risk factors

## Abstract

**Background:**

There are few reports on total parenteral nutrition (TPN) and its possible prothrombotic effect. The purpose of this study was to identify risk factors for subclavian vein thrombosis (SVT) in patients receiving TPN.

**Method:**

Cancer patients with indwelling subclavian catheters and TPN were followed in a cohort study. Doppler ultrasound examination was performed 8 and 30 days after catheter placement.

**Results:**

One hundred twenty-one patients were included, with a mean of 61 (± 11.8) years of age. We detected 36 SVT events at day 8 (29.8%) and 47 (38.8%) at day 30 after central catheter placement. Mean length of subclavian catheterization was 17.2 (± 8.2) days. Fifty-three point three percent of patients receiving ≥ 3,050 mOsm TPN in 24 hours developed SVT (relative risk (RR) = 2.01, 95% CI, 1.14 - 3.57; P = 0.016) at day 8 and 60% (RR = 1.67, 95% CI, 1.30 - 2.71; P = 0.038) at day 30 post-catheter placement. Protein administration of > 97.5 g/day was shown to be a risk factor for early thrombosis with a mean of 16.88 days for the development of SVT (95% CI, 10 - 23.7) versus 27.8 days (95% CI, 25.8 - 29.9) in the group with nutritional protein content < 97.5 g/day (P = 0.000).

**Conclusion:**

High-osmolarity and high-protein nutrition formulas were shown to be risk factors for SVT in cancer patients receiving TPN.

## Introduction

Venous thrombosis is a frequent complication in hospitalized cancer patients, although subclavian vein thrombosis (SVT) is not commonly diagnosed, with trauma and venous catheterization being its main causes. Long-term venous catheterization is common in cancer patients in order for them to receive treatments such as chemotherapy or parenteral nutrition. Central veins are preferred in this setting due to a reduced risk of infections and thrombosis compared with peripheral veins [1]. There is great variability in the cumulative incidence of thrombosis reported in patients with central indwelling catheters, ranging from 12 to 66% [2, 3]; usually, these events are asymptomatic (71-90%). Trials with the highest cumulative incidences have been conducted using contrast media and invasive diagnostic procedures. Previous cohort studies had established the onset of SVT between day 8 (64-98%) and day 30 after catheter insertion (68-98%) [4-6]. The main risk factors proposed as SVT etiology include infections, older age, metastatic cancer, previous thrombosis events, etc. [7, 8]. Little is known about total parenteral nutrition (TPN) and high osmolarity venous infusions as risk factors for venous thrombosis, although in animal models, a potential prothrombotic effect of parenteral nutrition was previously shown [9].

Doppler ultrasound has high sensitivity (78-94%) and specificity (82-96%) for the diagnosis of SVT [10, 11]; being a non-invasive test, and safer than venography, it is known as the diagnostic gold standard. Our objective in this study was to find out the cumulative incidence of SVT in patients on parenteral nutrition and to identify risk factors associated with this complication.

## Materials and Methods

Consecutive cancer patients requiring subclavian vein catheterization in order to receive TPN were included. All patients were receiving thromboprophylaxis with subcutaneous enoxaparin (1 mg/kg/day). Patients with previous subclavian catheter placement or previous thrombotic events were excluded.

### Study design

This was a prospective cohort study. A subclavian vein Doppler ultrasound (HDI 5000 Philips ATL broadband linear transducer, 12 to 5 MHz) was performed 8 (± 1) and 30 days (± 1) after catheter placement looking for venous thrombosis. By direct interviews, we collected data about age, comorbidities (diabetes, hypertension, autoimmune disorders, etc.) and smoking index. From medical records, we obtained data on type of cancer, body mass index (BMI), TPN formula received (osmolarity, calories, amounts of protein, carbohydrates and lipids), side and complications during catheter insertion, coagulation tests and catheter tip cultures. Our dependent variable, SVT, was defined as total or partial obstruction by a thrombus diagnosed by Doppler ultrasound examination. The study was carried out at the Oncology Hospital, Centro Medico Siglo XXI, IMSS, one of the main cancer reference centers in Mexico.

### Statistical analysis

Chi-square or Mann-Whitney U tests were performed depending on the type of variable. Relative risks (RRs) were established for variables with statistical significance. A Kaplan-Meier test was employed to analyze significant variables eliminating confounding factors, and a Cox proportional hazards model was used to establish the relationship between the cumulative incidence of thrombosis events and the number of days elapsed after catheter placement. Multiple logistic regression analyses helped us to establish a possible relationship between significant variables and SVT. A P < 0.05 was considered statistically significant. IBM SPSS Statistics 20 software was employed for statistical analysis.

### Ethical aspects

The study was considered to be a minimal risk research; it was reviewed and accepted by the Investigation Committee at the Oncology Hospital, Centro Medico Nacional Siglo XXI, in compliance with internationally accepted ethical codes and guidelines. All study subjects gave their written informed consent.

## Results

**Figure 1:**
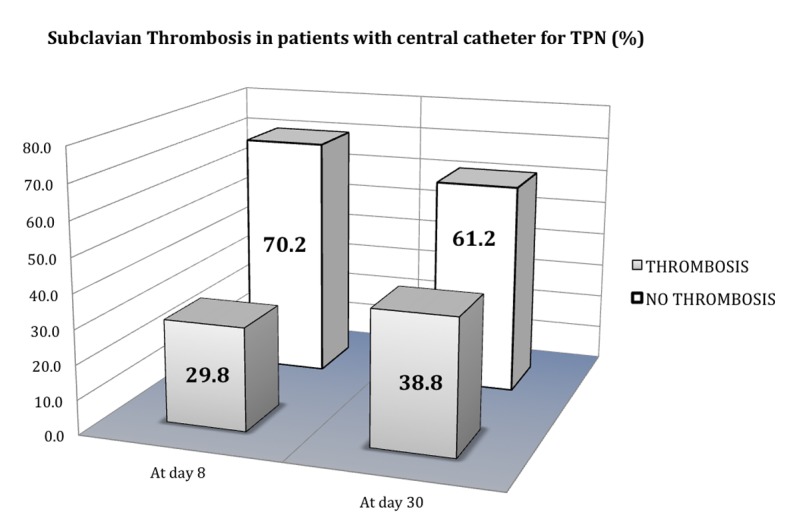
Subclavian vein thrombosis incidence in patients with central catheter for TPN.

From January 2012 to February 2013, 121 patients were included, 63 male (52.1%) and 58 female (47.9%), with a mean of 61 (± 11.8) years of age. The most common cancer locations were colorectal (37.2%), stomach (29.8%) and pancreas (19%). Thirty-six thrombosis events were detected at day 8 from catheter insertion for TPN (29.8%) and 47 at day 30 (38.8%) (Fig. 1).

Only four patients (3.3%) showed SVT-related symptoms, mainly ipsilateral arm edema. No total subclavian vein occlusion was reported, only partial events. No deep vein thrombosis events were recorded during the study.

At hospital admission, 56.2% of the subjects had normal weight, 29.8% were obese or overweight and 14% had low weight. Subclavian catheter was placed at the right side in 85.1% of the cases with a mean duration of 17.2 days of venous catheterization (95% CI, 15.71 - 18.67); parenteral nutrition was administered for 14.9 days (95% CI, 13.48 - 16.46); cumulative incidence of catheter colonization was 13.2% (Table 1); mean osmolarity of TPN solution administered was 2,526 (± 445) mOsm/day (Table 2).

**Table 1:** General Characteristics of the Study Population

Patients	121 (100%)
Male	63 (52.1%)
Age (years)	61 (± 11.8)*
Body mass index (kg/m^2^)	24.06 (± 4.5)*
Obesity (BMI > 30 kg/m^2^)	33 (29.7%)
Low weight (BMI < 20 kg/m^2^)	15 (13.5%)
Type of cancer	
Colorectal	45 (37.2%)
Gastric	36 (29.8%)
Pancreas	23 (19%)
Bladder	7 (5.8%)
Lymphoma	7 (5.8%)
Ovarian	3 (2.5%)
Comorbidities	
Diabetes mellitus	19 (15.7%)
Systemic hypertension	37 (30.6%)
COPD	17 (14%)
Renal disease	6 (5%)
Smokers	43 (35.5%)
Right subclavian catheter	103 (85.1%)
Catheter colonization	17 (14%)
Fibrinogen (mg/dL)	580 (± 212)*
Days with indwelling catheter	17.2 (± 8.2)*
Days with TPN	14.9 (± 8.2)*

*Mean (± SD).

**Table 2:** Total Parenteral Nutrition Formula Characteristics

Nutrients/characteristics	Mean (± SD)
Lipids (g/24 h)	49.17 (± 10.1)
Glucose (g/24h)	217.56 (± 37.7)
Proteins (g/24 h)	79.2 (± 17.8)
Osmolarity in 24 h (mOsm)	2,526 (± 445)
Osmolarity/liter (mOsm/L)	1,458 (± 79.3)
Calories/ideal body weight (kcal/kg)	26.8 (± 4.7)

Patients with gastric cancer showed more cumulative incidence of thrombosis compared with other types of cancer, with 38.9% at day 8 (RR = 1.5, 95% IC, 0.87 - 2.59; P = 0.11) and 50% at day 30 (RR = 1.46, 95% IC, 0.94 - 2.27; P = 0.076).

Mann-Whitney U test was employed to analyze quantitative variables. None of the previously reported possible risk factors for thrombosis showed significant differences (age, BMI, smoking index, time from catheter insertion), nor did the mean characteristics of the nutritional formula administered; only fibrinogen showed differences when thrombosis patients were compared with the non-thrombosis group: 438 vs. 585 mg/dL (P = 0.002) at day 8 and 446 vs. 613 mg/dL (P = 0.000) at day 30, respectively.

**Figure 2:**
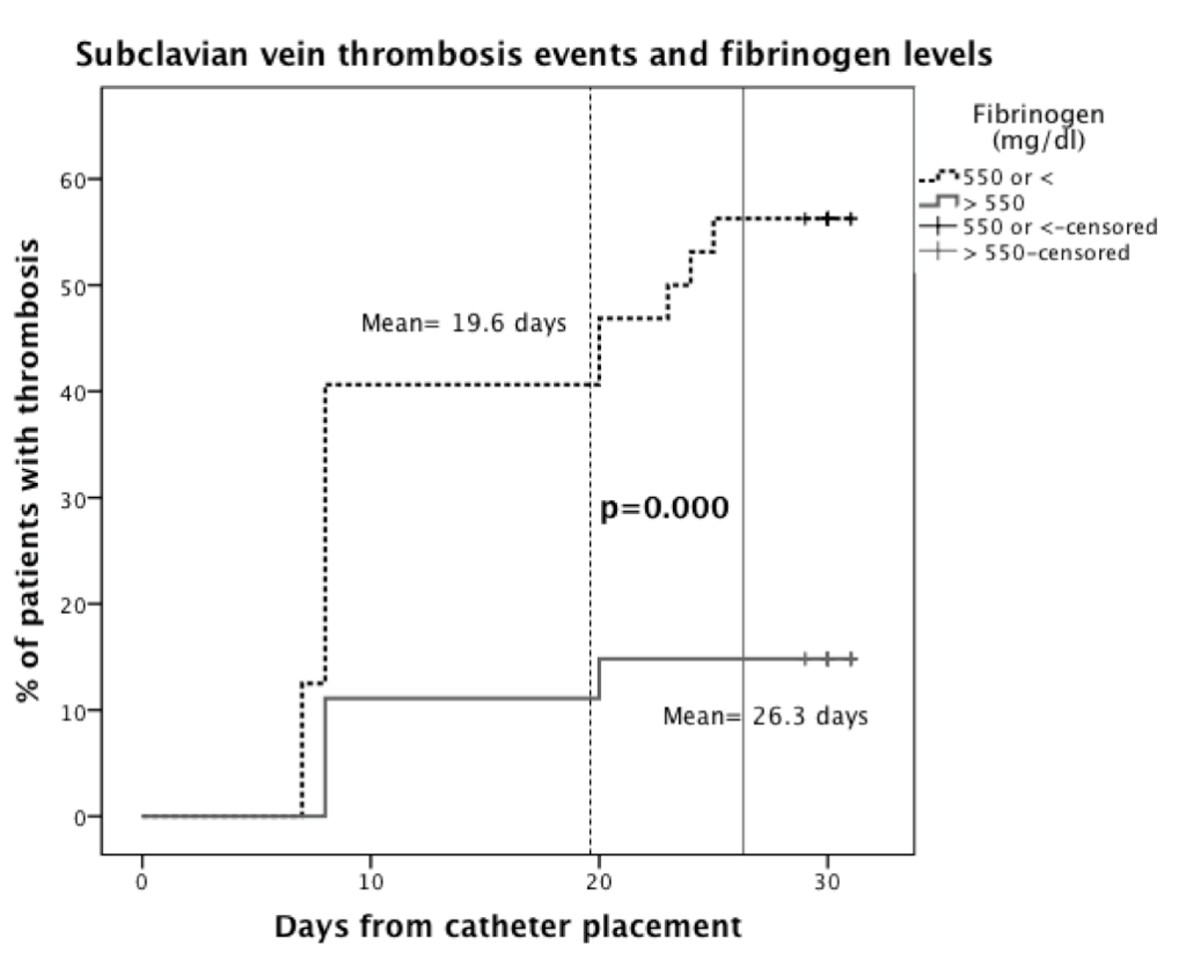
Kaplan-Meier plot. Time for the development of subclavian vein thrombosis according to fibrinogen levels.

In view of this finding, we tried to find a cutoff point in order to establish the risk of thrombosis according to the levels of fibrinogen. By means of an ROC analysis, we found that fibrinogen levels less than or equal to 550 mg/dL had an area under the curve (AUC) of 0.675 (0.571 - 0.78), P = 0.002, with a sensitivity of 66.7% and specificity of 61.2% for thrombosis at day 8, and an AUC of 0.745 (0.655 - 0.834), P = 0.000, with a sensitivity of 70% and specificity of 67.6% for thrombosis at day 30. A Kaplan-Meier analysis showed that this effect was related to the time for the development of SVT, with a mean of 19.6 (95% CI, 16.8 - 22.4) days in patients with fibrinogen levels < 550 mg/mL and a mean of 26.3 (95% CI, 24.1 - 28.5) days in patients with fibrinogen > 550 mg/mL (P < 0.000) (Fig. 2).

By means of a Chi-square test, we analyzed dichotomous variables, whereas for previously categorized quantitative variables, we used an ROC curve approach. At day 8, the variables with statistical significance were TPN formula total osmolarity higher than 3,050 mOsm/day with 53.3% of thrombosis events (RR = 2.01, 95% CI, 1.14 - 3.5; P = 0.016) and fibrinogen ≤ 550 mg/dL with 42.1% of thrombosis events (RR = 2.24, 95% CI, 1.2 - 4.06; P = 0.008). At day 30, both variables remained significant, with osmolarity higher than 3,050 mOsm/day being associated with 60% of thrombosis events (RR = 1.67, 95% CI, 1.3 - 2.71; P = 0.038), and fibrinogen ≤ 550 mg/dL with 57.9% of thrombosis events (RR = 2.64, 95% CI, 1.58 - 4.42; P = 0.000).

**Figure 3:**
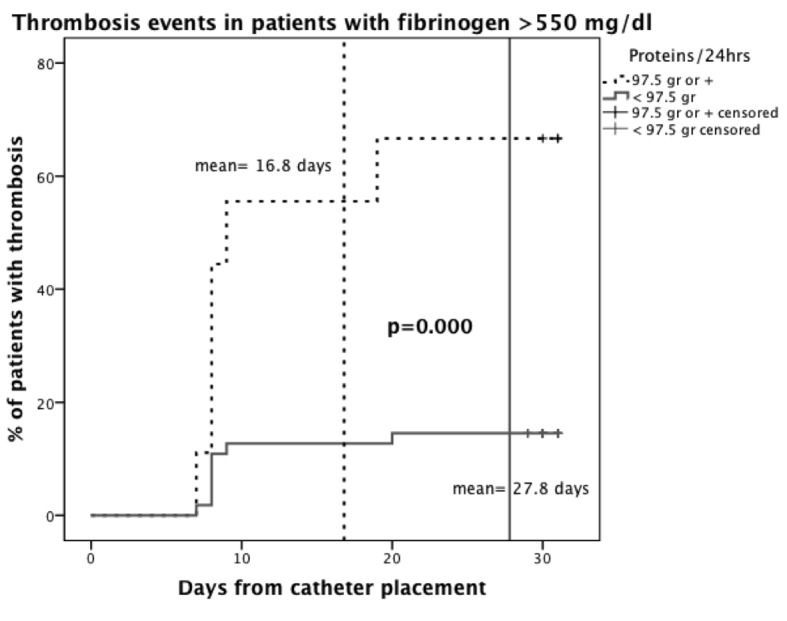
Kaplan-Meier plot. Subclavian vein thrombosis in patients with fibrinogen > 550 mg/dL according to protein administration in daily TPN.

A Kaplan-Meier curve was plotted to analyze the independent variables without the possible bias of normal to low fibrinogen levels. Protein administration of 97.5 g/day or more showed an effect on the risk of thrombosis in patients with high fibrinogen levels (> 550 mg/dL), with a mean of 16.88 days to develop thrombosis (95% CI, 10 - 23.7) versus 27.8 days (95% CI, 25.8 - 29.9) in the group with < 97.5 g/day nutritional protein content, (P = 0.000) (Fig. 3).

**Figure 4:**
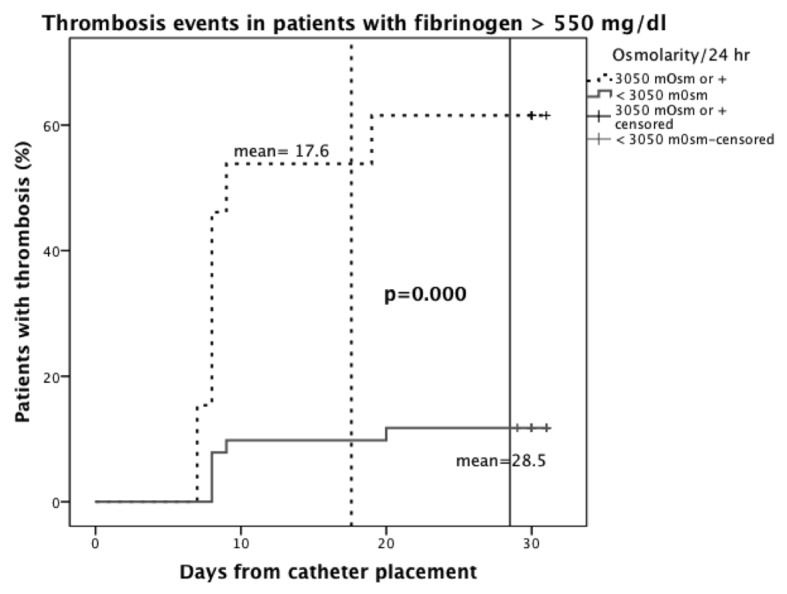
Kaplan-Meier plot. Thrombosis in patients with fibrinogen > 550 mg/dL distributed by osmolarity in daily TPN formula received.

In the high-fibrinogen group, TPN with osmolarity > 3,050 mOsm/day resulted in a mean of 17.6 days for the development of thrombosis (95% CI, 11.6 - 23.5) versus 28.5 (95% CI, 26.6 - 30.4) days in the group with < 3,050 mOsm/day TPN (P = 0.000) (Fig. 4).

**Figure 5:**
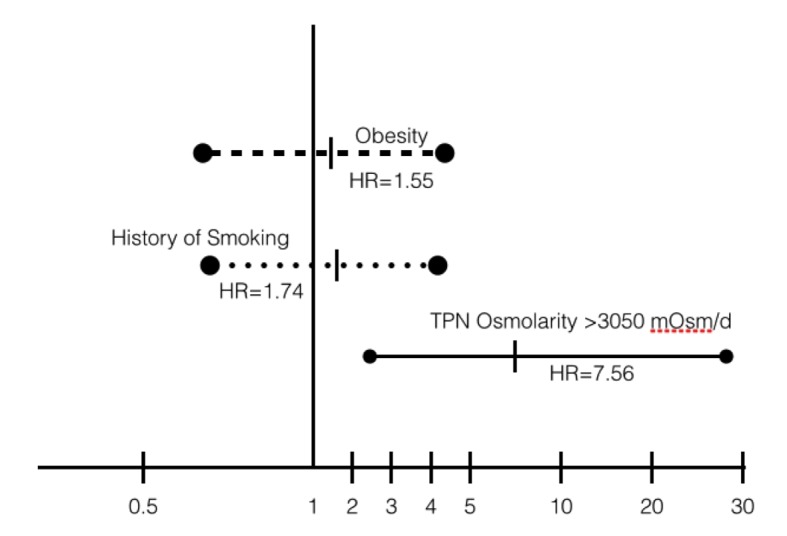
Forrest plot of HRs for venous subclavian vein thrombosis at day 8 from the start of TPN.

**Figure 6:**
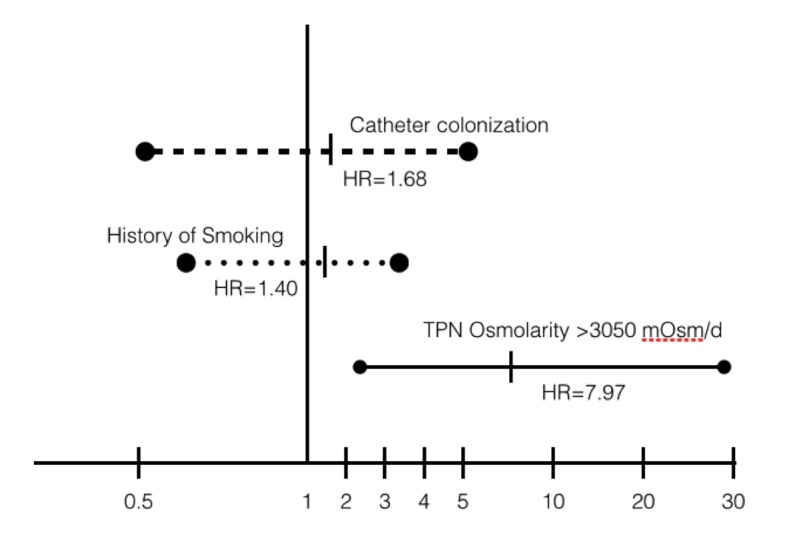
Forrest plot of HRs for venous subclavian vein thrombosis at day 30 from the start of TPN.

By means of multiple logistic regression analyses, we found that TPN formulas with osmolarity over 3,050 mOsm/day (hazard ratio (HR) = 7.56; 95% CI, 2.04 - 28.01; P = 0.002 at day 8, and HR = 7.97; 95% CI, 2.15 - 29.53; P = 0.002 at day 30) and fibrinogen levels < 550 mg/dL (HR = 5.45; 95% CI, 2.02 - 14.7; P = 0.001 at day 8, and HR = 8.46; 95% CI, 3.27 - 21.8; P = 0.000 at day 30) were significant predictors of subclavian thrombosis with a global effect of up to 71.1% at day 30 (-2log likelihood = 134.4, r^2^ = 0.201). HRs for subclavian venous thrombosis and high osmolarity formulas were compared in a Forrest plot against previously known risk factors mentioned in literature (Fig. 5, 6).

**Figure 7:**
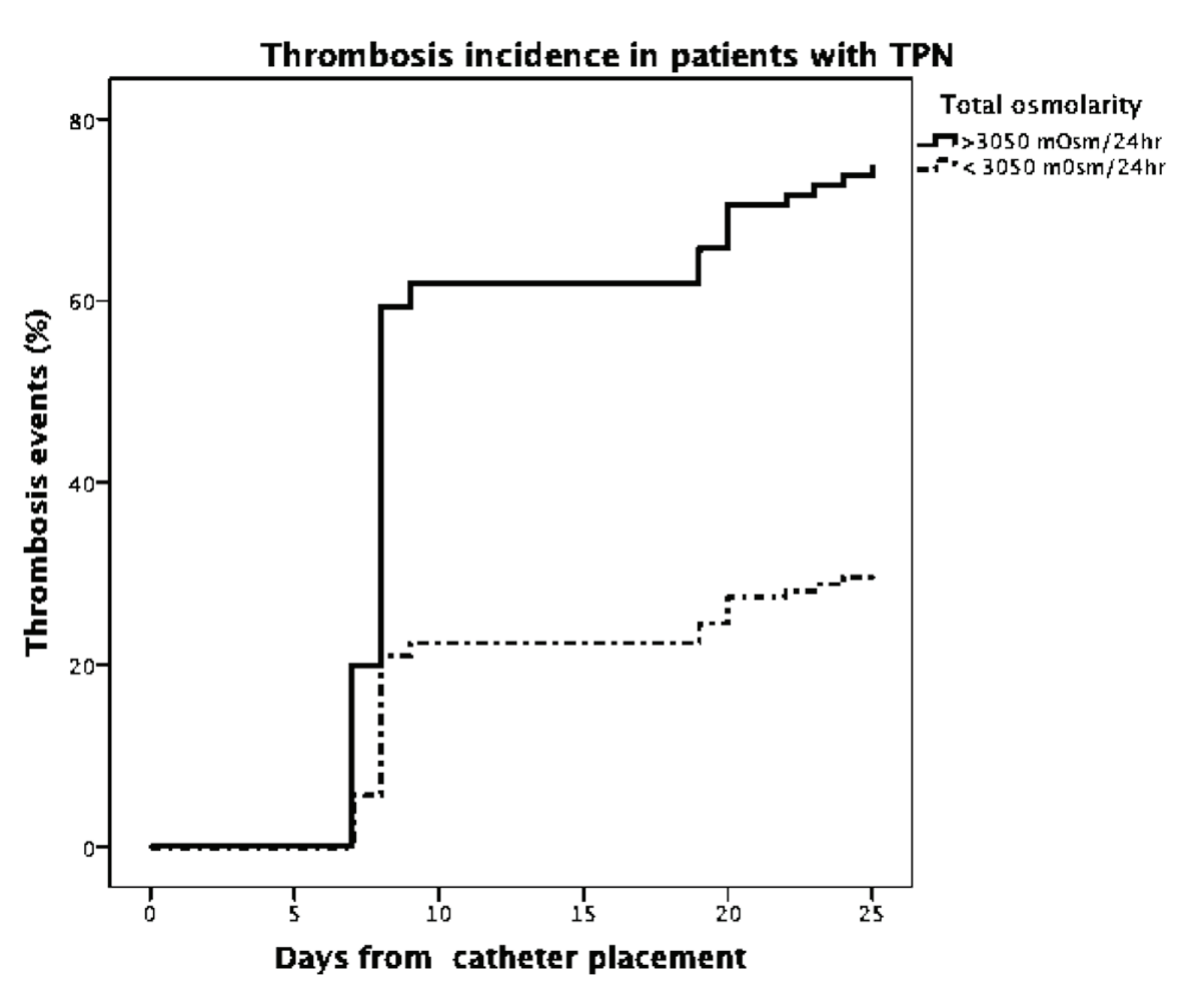
Cox regression analysis. Thrombosis events by days after catheter placement distributed by osmolarity of TPN received in 24 h.

Using a Cox regression analysis, we showed the difference in the cumulative incidence of SVT when patients received high-osmolarity TPN formulas (HR = 3.6, 95% CI, 1.68 - 8.09; P = 0.001) (Fig. 7).

## Discussion

High cumulative incidence of SVT was found in high-risk patients with cancer, recent surgery and parenteral nutrition, in spite of antithrombotic prophylaxis.

This incidence is comparable to that in previous reports in non-oncologic patients receiving TPN without thrombotic prophylaxis [12]. Symptomatic events showed similar incidence as in previously reported studies with non-radiologic diagnosis of thrombosis (3.3%). Most thrombosis events were observed at day 7 from catheter placement (76.5%), similar as the 64% reported by De Cicco and colleagues using flebography in cancer patients [13].

We included clinical variables previously shown to be risk factors for thrombotic events [8], adding the nutritional formula elements and characteristics. No significant differences were found in age group, presence of bacterial colonization, puncture side or complications during catheter placement. A higher incidence of subclavian thrombosis was found in gastric cancer patients; there are previous reports of C-protein-acquired resistance leading to a hypercoagulable state [14, 15]. None of these patients had known metastases at the time of the study.

Analyzing the nutritional formula characteristics, we found that high amino acid concentrations (> 97.5 g) and high osmolarity (> 3,050 mOsm) administered in 24 h were related to SVT with statistical significance.

We included laboratory parameters not previously considered as being related to SVT. In this study, fibrinogen levels < 550 mg/dL were found to be a risk factor for subclavian thrombosis; this is an element of the coagulation cascade that at high levels is associated with atherosclerosis and prothrombotic states [16]; whereas low levels are related mainly with disseminated intravascular coagulation. In subjects with systemic inflammatory responses and in cancer patients, a 200 to 400% increase in fibrinogen levels is expected [17]; in our study, we found a significant relationship between subclavian thrombosis events and normal to 50%-higher levels of fibrinogen. Some studies had found coagulation tests anomalies in patients with cervix [18], lung [19, 20], ovarian [21] or breast cancer [22]. We think that this finding is likely to be related to a pro-inflammatory effect rather than a risk factor.

This study had some limitations. First, our patients were not assessed with an initial ultrasound, they were asymptomatic prior to the catheterization, and we considered that the only way to install a venous catheter was using a permeable vein. Another limitation was that D-dimer levels were not measured, as this is not a routine laboratory test in our hospital because levels are considered to be usually high in cancer patients. The present study gave us some insights on the incidence of this complication and possible risk factors. We propose close monitoring of venous patency in patients receiving high-osmolarity TPN formulas, and we also hope that this report will provide grounds for new studies on this topic.

### Conclusion

High-osmolarity and high-protein nutrition formulas were shown to be risk factors for SVT in cancer patients receiving TPN. We propose close monitoring in high-risk patients with Doppler ultrasound testing 30 days after catheter insertion for prompt treatment.
